# Electrogenerated Chemiluminescence Behavior of Au nanoparticles-hybridized Pb (II) metal-organic framework and its application in selective sensing hexavalent chromium

**DOI:** 10.1038/srep22059

**Published:** 2016-02-23

**Authors:** Hongmin Ma, Xiaojian Li, Tao Yan, Yan Li, Haiyang Liu, Yong Zhang, Dan Wu, Bin Du, Qin Wei

**Affiliations:** 1 Key Laboratory of Chemical Sensing & Analysis in Universities of Shandong, School of Chemistry and Chemical Engineering, University of Jinan, Jinan 250022, PR China; 2 School of Resources and Environment, University of Jinan, Jinan 250022, PR China

## Abstract

In this work, a novel electrochemiluminescence (ECL) sensor based on Au nanoparticles-hybridized Pb (II)-β-cyclodextrin (Pb-β-CD) metal-organic framework for detecting hexavalent chromium (Cr(VI)) was developed. Pb-β-CD shows excellent ECL behavior and unexpected reducing ability towards Au ions. Au nanoparticles could massively form on the surface of Pb-β-CD (Au@Pb-β-CD) without use of any additional reducing agent. In the presence of coreactant K_2_S_2_O_8_, the ECL emission of Pb-β-CD was enhanced by the formation of Au nanoparticles. Cr(VI) can collisionally quench the ECL behavior of Au@Pb-β-CD/S_2_O_8_^2−^ system and the detection mechanism was investigated. This ECL sensor is found to have a linear response in the range of 0.01–100 μM and a low detection limit of 3.43 nM (S/N = 3) under the optimal conditions. These results suggest that metal-organic framework Au@Pb-β-CD has great potential in extending the application in the ECL field as an efficient luminophore.

Chromium is widely used in industrial processes such as metals melting, leather tanning, electroplating and dyestuff, where huge volumes of waters containing hexavalent chromium (Cr(VI)) are generated in many rinsing operations^1^. In aqueous solutions, chromium mainly exists in two valence states; namely trivalent chromium Cr(III) and Cr(VI)^2^. Cr(III) is considered to have low toxicity and is an essential trace element for mammals, while Cr(VI) is approximately 100 times more toxic and is considered a human carcinogen with adverse impact on human skin, stomachs, lung, liver and kidneys^3^,^4^,^5^. In addition, chromium can also enter drinking water supply systems via corrosion inhibitors used in water pipes and containers or via contamination of underground water leaching from sanitary landfill^6^,^7^. And many conventional techniques, such as ion-exchange and membrane processes, were used to remove Cr(VI) until the concentration of Cr(VI) less than 100 mg mL^−1^ allowing water reuse^1^,^4^. Therefore, developed a simple analytical method for detecting trace concentration Cr(VI) is very important due to its high toxicity.

According to reported detecting methods, such as inductively coupled plasma mass spectrometry^8^, inductively coupled plasmaoptical emission spectrometry^9^, inductively coupled plasma atomic emission spectrometry^10^, electrothermal atomic absorption spectrometry^3^, graphite furnace atomic absorption spectrometry^11^, reversed-phase ion-pair chromatography^12^, X-ray fluorescence spectrometry^13^, ECL spectrometry^14^, and stripping voltammetric methods^15^,^16^, they were used to detect the total quantity of chromium. In this work, a new and simple electrochemiluminescence (ECL) sensor based on Au nanoparticles- hybridized Pb (II) metal-organic framework was developed to only detect Cr(VI).

Metal-organic frameworks (MOFs) have attracted extensive research interests, especially in the fields of ion exchange, gas storage and separation^17^, sensing probe and catalysis^18^,^19^. As a special type of porous materials, cyclodextrins (CDs)-based MOFs have recently attracted considerable attention attributing to its special structure and properties. CDs have a large number of glycosidic oxygen atoms and can provide plenty of coordination sites to chelate metal ions^20^. Moreover, the water solubility and biocompatibility of CDs^21^ make CDs-based MOFs as good candidates for application in biological sensing filed. Therefore, we report the ECL behavior of Pb-β-CD using K_2_S_2_O_8_ as a coreactant in the present work and Pb-β-CD shows excellent ECL behavior. In quenching ECL system, it is important to increase the ECL intensity of substrate luminescent materials. Thus, Au nanoparticles were immobilized on the surface of Pb-β-CD to enhance the ECL intensity of Pb-β-CD and improve the sensitivity of ECL sensor^22^. What’s more, Pb-β-CD shows unexpected reducing capacity towards AuCl_4_^−^, so AuCl_4_^−^ was reduced to Au nanoparticles without adding any reductant. Then, Au nanoparticles hybridized Pb-β-CD (Au@Pb-β-CD) was prepared by a simple method and was used to fabricate an ECL sensor.

In this work, a new type ECL sensor for detecting Cr(VI) was developed using the as-prepared Au@Pb-β-CD as substrate luminescent materials. The detection mechanism was that Cr(VI) can quench the strong cathodic ECL signal of Au@Pb-β-CD/S_2_O_8_^2−^ system. This work extends the applications of CDs-based MOFs and provides a versatile avenue for selective and sensitive detection of Cr(VI).

## Experimental Section

### Materials

HAuCl_4_·6H_2_O, β-CD, K_2_Cr_2_O_7_, PbCl_2_ and β-cyclohexanol were purchased from Shanghai Chemical Reagent Co. Ltd., (Shanghai, China). All other chemicals were of analytical reagent grade and were used without further purification. Phosphate buffer saline (PBS) was prepared by using 1/15 mol ∙ L^−1^ Na_2_HPO_4_ and 1/15 mol ∙ L^−1^ KH_2_PO_4_ solution. K_3_Fe(CN)_6_/K_4_Fe(CN)_6_ (2.5 mM) and KCl (0.1 M) solution were used as electrolyte for electrochemical impedance spectroscopy (EIS). All aqueous solutions were prepared using ultrapure water. To eliminate the interference, 0.1 mM EDTA was added into the above solution which contained 2 μM metal ions (Pb^2+^, Fe^3+^, Zn^2+^, Cr^3+^, Cd^2+^, Co^2+^and Cu^2+^), 0.1 M K_2_S_2_O_8_ and 0.1 M KCl solution.

### Apparatus

Transmission electron microscope (TEM) images were obtained from a JEM-2100 microscope (Japan). Scanning electron microscope (SEM) and Energy Dispersive X-Ray Spectroscopy (EDX) were recorded by JEOL JSM-6700F microscope (Japan). The ECL measurements were performed with a MPI-F chemiluminescence detector (Xi’an remax Electronic Science Tech. Co. Ltd., China) and electrochemical measurements were carried out on CHI760D electrochemical workstation (Chenhua Instrument Shanghai Co., Ltd, China) using a three-electrode system consisted of a platinum wire as an auxiliary electrode, an Ag/AgCl electrode as reference electrode, and a glassy carbon electrode (GCE, 4 mm in diameter) as working electrode.

### Preparation of Au@Pb-β-CD

Pb-β-CD was prepared as previously described with some slight modifications^20^. 23 mg β-CD and 45 mg PbCl_2_ were dispersed in 6 mL ultrapure water. The mixture was stirred at room temperature. Then, the mixture was transformed to reaction kettle, and 6 mL mixed solvent of cyclohexanol and triethylamine (v/v = 1:1) was slowly added. The reaction kettle was then sealed and heated to 110 °C for 3 days. The finally product was washed with ultrapure water and dried in air to obtain Pb-β-CD.

Au@Pb-β-CD was synthesized as follows: 100 mg Pb-β-CD was dispersed in 50 mL ultrapure water by ultrasonication for 4 h. Then, 2 mL 2% HAuCl_4_·6H_2_O was added in the above solution and stirred overnight, followed by centrifuging to remove unreacted HAuCl_4_. The finally product was vacuum dried at 35 °C for 12 h. The obtained Au@Pb-β-CD was dispersed in 0.5% chitosan until use.

### Preparation and measurement procedure of ECL sensor

**Figure 1:**
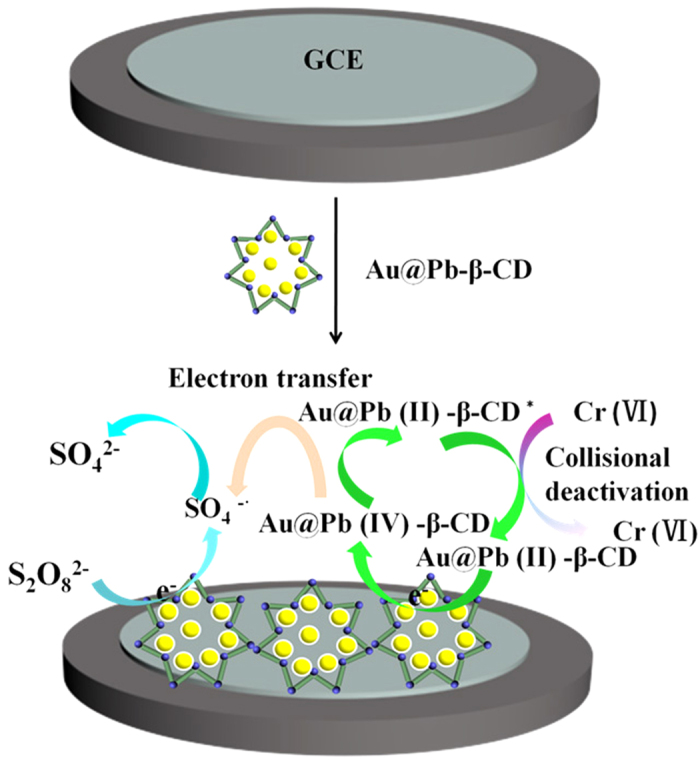
Schematic diagram for the fabrication of the sensor.

Figure 1 shows the schematic diagram for the fabrication of an ECL sensor for the detection of Cr(VI). GCE with 4 mm diameter was polished to a mirror-like finish with 1.0, 0.3 and 0.05 μm alumina powder and then thoroughly cleaned before use. Then 10 μL 10 mg/mL Au@Pb-β-CD was dropped onto the electrode surface and dried at room temperature. Finally, the ECL signal was detected in pH 7.4 PBS containing different concentrations of Cr(VI). The scanning potential was −1.6–0 V and the photomultiplier tube (PMT) was set at 800 V. Scan rate: 0.05 V s^−1^.

## Results and Discussion

### Characterization of Pb-β-CD and Au@Pb-β-CD

**Figure 2:**
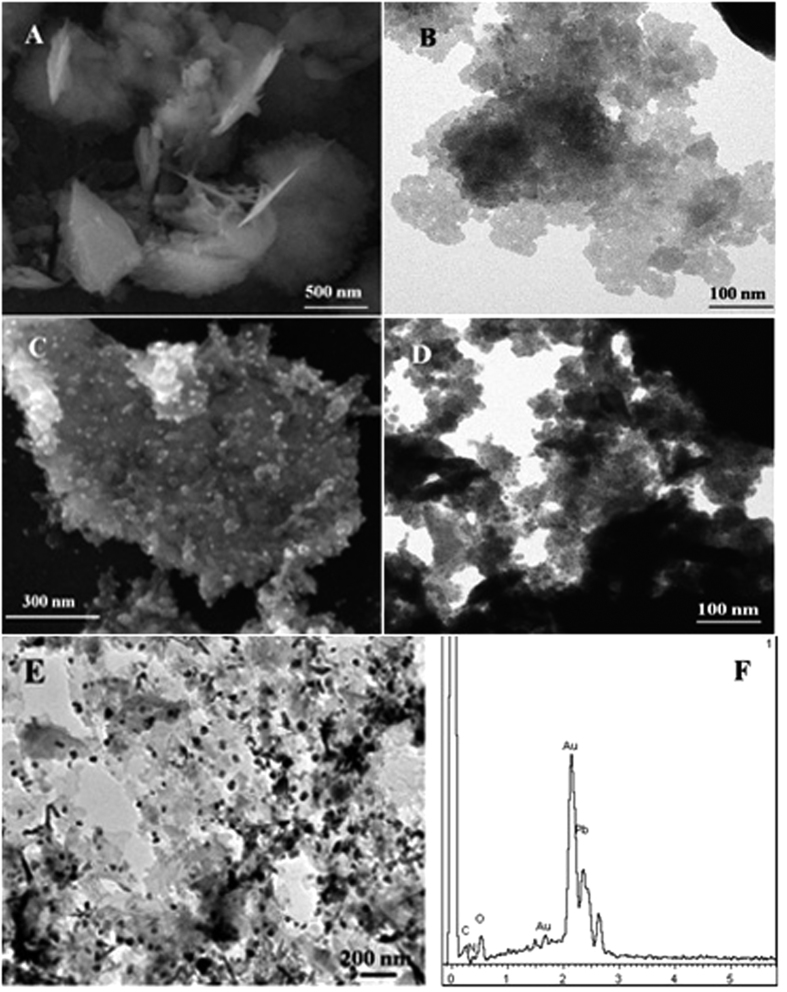
SEM (**A**) and TEM (**B**) image of Pb-β-CD; SEM (**C**) and TEM (**D**) image of Au@Pb-β-CD; high resolution HR-TEM image (**E**) and EDX (**F**) of Au@Pb-β-CD.

Figure 2A,B shows the SEM and TEM images of the prepared Pb-β-CD, illustrating that Pb-β-CD was porous and flake-like shaped metal-organic framework. Figure 2C,D shows that Au nanoparticles were dispersed on the surface of Pb-β-CD (Au@Pb-β-CD). Figure 2E was the high resolution HR-TEM image of Au@Pb-β-CD and Au NPs was clearly seen to immobilized on the surface of Pb-β-CD. EDX was also used to investigate the composition of Au@Pb-β-CD. From Fig. 2F, we can see Au@Pb-β-CD had been synthesized successfully.

### Mechanism of Au@Pb-β-CD ECL emission and the quenching mechanism toward Cr(VI)

The ECL behavior of Au@Pb-β-CD mainly caused by Pb-β-CD and Au NPs can enhance the ECL intensity of Pb-β-CD. Analogous to the ECL pathways of Ru(bpy)_3_^2+^, the ECL excitation route of Pb-β-CD was the oxidation-initiated reductive excitation pathway^23^,^24^. The whole process could be stated as follows:


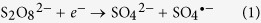



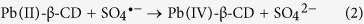



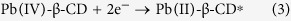



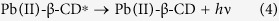


**Figure 3:**
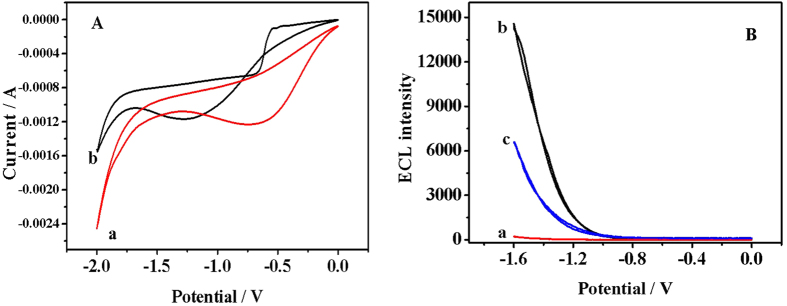
(**A**) CV of bare GCE (curve a) and Pb-β-CD-modified GCE (curve b); (**B**) ECL-potential curves of Pb-β-CD (a), Au@Pb-β-CD in the absence (b) and presence (c) of Cr(VI).

Figure 3A displays that a broad peak at ca. −0.72 V (vs Ag/AgCl) is found for bare GCE with K_2_S_2_O_8_ (curve b). It should be S_2_O_8_^2−^ is electro-reduced to SO_4_^•−^ and SO_4_^2^ (eq1)^25^^–^27 Similar CV scans were conducted on the Pb-β-CD-modified GCE with K_2_S_2_O_8_ (curve a). A broad reduction peak of S_2_O_8_^2−^ at ca.−1.3 V (vs. Ag/AgCl) is observed which is more negative than that of the bare GCE. The strong oxidant species (SO_4_^•−^) subsequently reacted with Pb(II)-β-CD to produce Pb (IV)-β-CD (eq2). Then, electrons from the working electrode is injected to Pb (IV)-β-CD producing the excited state Pb(II)-β-CD^*^ (eq3). When the excited state Pb(II)-β-CD^*^ jumped back to the ground state Pb(II)-β-CD, the ECL intensity can be detected (eq4).

Figure 3B also displays the ECL-potential curves of the Au@Pb-β-CD-modified GCE in the absence (curve b) and presence (curve c) of Cr(VI) by cycling the potential from −1.6 to 0 V. The strong ECL signal can be sensitively quenched by Cr(VI) in neutral aqueous solution. Like PL signal, the ECL signal can be quenched through static or dynamic avenues. Static avenues mean the quencher react with either the luminophore or the coreactant. Apparently, the quenching efficient should be affected by the concentrations of either the luminophore or the coreactant if the ECL signal of Au@Pb-β-CD was quenched by Cr(VI) through a static avenue. It has been reported that heavy atoms can collisionally deactivate the excited-state of luminophores, resulting the quenching of PL signal. Then the ECL quenching of Au@Pb-β-CD by Cr(VI) might undergo the same collisional deactivation process. In other words, Au@Pb-β-CD^*^ is deactivated upon contact with Cr(VI), leading to the ECL intensity decrease.

### Optimization of experimental conditions

**Figure 4:**
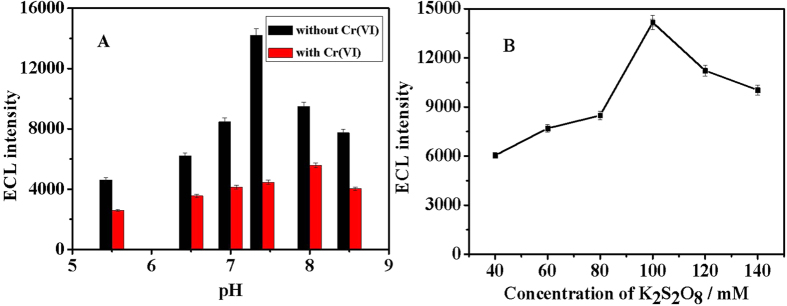
The effect of pH (**A**) on the response of ECL intensity from Au@Pb-β-CD/GCE without Cr(VI) (black column) and with 500 nM Cr(VI) (red column); The effect of concentration of K_2_S_2_O_8_ (**B**) on the response of ECL intensity from Au@Pb-β-CD/GCE. Error bars = RSD (*n* = 3).

The pH value of solution is one important factor for the sensing platform. It affects not only the ECL intensity of Au@Pb-β-CD, but also the quenching efficiency of Cr(VI) for the ECL of Au@Pb-β-CD. The influence of pH from 5.5 to 8.5 on the sensor performance was investigated. As described in Fig. 4A black column, the maximum ECL response appeared at pH 7.4. From Fig. 4A red column, we can see that when pH was at 7.4, the quenching efficiency of Cr(VI) toward the ECL signal of Au@Pb-β-CD was optimal. The effect of pH on the quenching efficiency may be related to the existing forms in the solution of Cr(VI). Cr(VI) exists in solution as either CrO_4_^2−^ or Cr_2_O_7_^2−^, depending on the pH value of the solution. In acidic solutions, Cr(VI) exists predominately as CrO_4_^2−^, which may has lower quenching efficiency towards Pb-β-CD^*^. Therefore, Cr(VI) can’t efficiently quench the ECL signal of Au@Pb-β-CD in acidic solutions. In alkaline solutions, the collision between Pb-β-CD^*^ and the negatively charged Cr_2_O_7_^2−^ was hardly occur. Therefore, pH 7.4 was chosen as the pH value of the detection solution.

The concentration of coreactant K_2_S_2_O_8_ was another important factor for the sensing platform. As shown in Fig. 4B, when the concentration of K_2_S_2_O_8_ was 100 mM, the ECL intensity of Au@Pb-β-CD achieved a maximum value. Because more Au@Pb-β-CD^*^ is produced from oxidation of the negatively charged Au@Pb-β-CD by the electrogenerated SO_4_^−^. Further increase in K_2_S_2_O_8_ concentration caused the decrease in ECL intensity as excess S_2_O_8_^2−^ would react readily with the negatively charged Au@Pb-β-CD which inhibited the formation of the excited-state Au@Pb-β-CD^* ^28.

### Performance of the sensor

**Figure 5:**
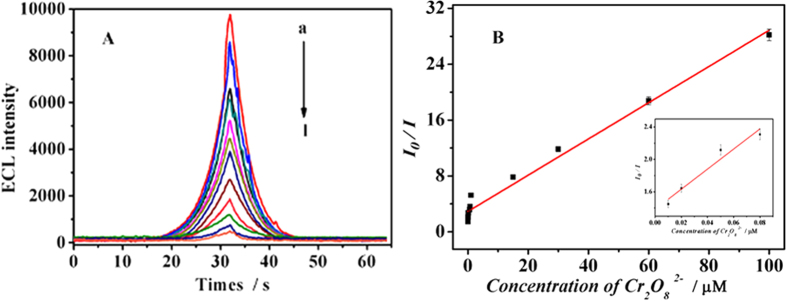
(**A**) ECL response of the sensor to different concentrations of Cr(VI), from a to l: 0.01, 0.02, 0.05, 0.08, 0.15, 0.5, 0.7, 1, 15, 30, 60, 100 μM;. (**B**) Calibration curve of the sensor for different concentrations of Cr(VI), the inset: calibration curve for lower concentrations of Cr(VI). Error bars = RSD (*n* = 3).

The analytical performance of the developed sensor was incubated with different concentrations of Cr(VI) solution (mainly existing as Cr_2_O_7_^2−^). Under the optimal assay condition, ECL intensity decreased linearly with the Cr(VI) concentration in the range of 0.01–100 μM (Fig. 5A, curves a-l). As shown in Fig. 5B, the linear equation was *I*_*0 *_*/I* = 0.259*c *+ 2.92 (where *I*_*0*_ *and I* is the ECL intensity of the absence and presence of Cr_2_O_7_^2−^ and *c* stands for the concentration of Cr_2_O_7_^2−^), with a correlation coefficient of 0.9822 and a detection limit of 3.43 nM (S/N = 3). A comparison for the performance of the proposed and referenced sensors for Cr(VI) detection have been provided. The analytical parameters including detection limit and linear range are comparable or better than the results reported for determination of Cr(VI), as displayed in Table 1. Therefore, the proposed ECL sensor showed higher sensitivity and wider linear range.

### Selectivity, stability and reproducibility

**Figure 6:**
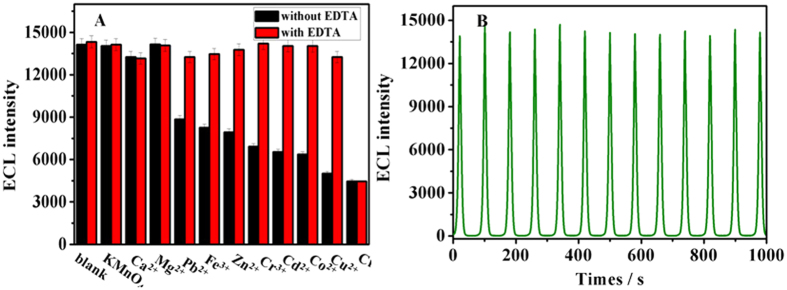
(**A**) Selectivity of the Au@Pb-β-CD/S_2_O_8_^2−^ system based sensor for Cr(VI) over other ions (KMnO_4_, Ca^2+^, Mg^2+^, Pb^2+^, Fe^3+^, Zn^2+^, Cr^3+^, Cd^2+^, Co^2+^, Cu^2+^, Cr_2_O_7_^2−^) in PBS containing 0.1 M KCl, 0.1 M K_2_S_2_O_8_, 2 μM all metal ions and 0.1 mM EDTA. (**B**) Stability of ECL emissions from Au@Pb-β-CD modified-GCE under continuous scanning for 13 cycles in PBS (pH = 7.4) containing 0.1 M KCl and 0.1 M K_2_S_2_O_8_. Error bars = RSD (*n* = 3).

The response of the proposed sensors to some common co-existing ions with Cr_2_O_7_^2−^ in samples was investigated. In the interference experiment, the substrate solution contained 2 μM interfering substances (including KMnO_4_, Ca^2+^, Mg^2+^, Pb^2+^, Fe^3+^, Zn^2+^, Cr^3+^, Cd^2+^, Co^2+^and Cu^2+^), 0.1 M K_2_S_2_O_8_ and 0.1 M KCl solution. The counter anions were NO_3_^−^ and Cl^−^, which were proved to have no obvious effect on the ECL of Au@Pb-β-CD/S_2_O_8_^2−^ system. Figure 6A black column displayed that many metal ions including Pb^2+^, Fe^3+^, Zn^2+^, Cr^3+^, Cd^2+^, Co^2+^and Cu^2+^can also quench the ECL signal of Au@Pb-β-CD/S_2_O_8_^2−^ system. Therefore, a suitable masking ligand is necessary to improve the selectively of the sensing system toward Cr(VI). EDTA is a well-known masking ligand which can complex most metallic cations. However, EDTA has nearly no effect on Cr(VI) due to that Cr(VI) exists mainly as Cr_2_O_7_^2−^ in the pH 7.4 solution. Accordingly, EDTA was added in the sensing system to effectively eliminate the interferences from other metal ions. As shown in Fig. 6 (red columns), the ECL signal was quenched by only Cr(VI) in the presence of 0.1 mM EDTA. The results indicated that the selectivity of the sensor was acceptable.

Excellent stability is one of the main points for extending potential application in the sensing field. Figure 6B displayed the ECL emission of the sensor under 13 cycles of continuous potential scans between −1.6–0 V in PBS (pH = 7.4) containing 0.1 M KCl and 0.1 M K_2_S_2_O_8_ at 50 mV s^−1^. The ECL intensity from Au@Pb-β-CD was pretty stable with the relative standard deviation of 2.02%, which indicated that the sensing signal was quite reliable.

The reproducibility of the sensor was also investigated by prepared seven electrodes for the detection 5 nM of Cr(VI). RSD of measurements was under 5%, indicating that the sensor had well reproducibility.

### Real samples analysis

To further investigate the potential application of the sensor for practical analysis, the sensor was used to test the recovery of different concentrations of Cr(VI) in river water samples. Different concentrations (1.00, 50.0 and 100.0 nM) of Cr(VI) spiked water samples were prepared by standard addition methods. The recovery of Cr(VI) was in the range of 95.0–101% and the RSD was in the range of 1.9–2.9% (Table 2). The results showed that the developed sensor might be preliminarily applied for the determination of Cr(VI) in real samples.

## Conclusion

To conclude, a simple, convenient and sensitive approach for determination of Cr(VI) has been shown. The ECL was first observed from Pb-β-CD which has excellent and stable ECL behaviour and the mechanism of Pb-β-CD/K_2_S_2_O_8_ system was investigated in detail. Moreover, Pb-β-CD has reducing capacity to reduce AuCl_4_^−^ and Au NPs can enhance the ECL intensity of Pb-β-CD. Cr(VI) was found to be able to quench the ECL signal of Au@Pb-β-CD/K_2_S_2_O_8_ system. This proposed method not only expands the application of metal-organic frameworks materials Pb-β-CD, but also opens new doors towards the detection of Cr(VI).

## Additional Information

**How to cite this article**: Ma, H. *et al.* Electrogenerated Chemiluminescence Behavior of Au nanoparticles-hybridized Pb (II) metal-organic framework and its application in selective sensing hexavalent chromium. *Sci. Rep.* **6**, 22059; doi: 10.1038/srep22059 (2016).

## Figures and Tables

**Table 1:** The developed ECL sensors for detecting Cr(VI) compared to other published sensor.

Method	Materials	linear range	detection limit	References
ECL	Graphene quantum dots	50 nM–60 μM	20 nM	^14^
Optical sensor	Anion-exchange membrane	10.197 mM–54.390 mM	–	^1^
Fluorescence	Glutathione-stabilized gold nanoclusters	0.0167–1.670 μM	1.6 nM	^2^
ECL	Au@Pb-β-CD	0.01–100 μM	3.43 nM	This work

**Table 2:** Results for Cr(VI) determination in samples.

Sample	Addition (nM)	Detection (*n* = 5, nM)	RSD (%)	Recovery (%)
River water	100.0	100.3	2.5	100.3
	50.0	50.35	1.9	100.7
	1.00	0.95	2.9	95.0
